# Creating a Long-Term Diabetic Rabbit Model

**DOI:** 10.1155/2010/289614

**Published:** 2010-12-27

**Authors:** Jianpu Wang, Rong Wan, Yiqun Mo, Qunwei Zhang, Leslie C. Sherwood, Sufan Chien

**Affiliations:** ^1^Department of Surgery, University of Louisville, MDR 316, Louisville, KY 40202, USA; ^2^Department of Environmental and Occupational Health Sciences, University of Louisville, Louisville, KY 40202, USA; ^3^Research Resources Facilities and Cardiovascular Innovation Institute, University of Louisville, Louisville, KY 40202, USA

## Abstract

This study was to create a long-term rabbit model of diabetes mellitus for medical studies of up to one year or longer and to evaluate the effects of chronic hyperglycemia on damage of major organs. A single dose of alloxan monohydrate (100 mg/kg) was given intravenously to 20 young New Zealand White rabbits. Another 12 age-matched normal rabbits were used as controls. Hyperglycemia developed within 48 hours after treatment with alloxan. Insulin was given daily after diabetes developed. All animals gained some body weight, but the gain was much less than the age-matched nondiabetic rabbits. Hyperlipidemia, higher blood urea nitrogen and creatinine were found in the diabetic animals. Histologically, the pancreas showed marked beta cell damage. The kidneys showed significantly thickened afferent glomerular arterioles with narrowed lumens along with glomerular atrophy. Lipid accumulation in the cytoplasm of hepatocytes appeared as vacuoles. Full-thickness skin wound healing was delayed. In summary, with careful management, alloxan-induced diabetic rabbits can be maintained for one year or longer in reasonably good health for diabetic studies.

## 1. Introduction

Diabetes Mellitus is one of the leading causes of death, illness, and economic loss in the United States [1]. Not surprisingly, an extensive amount of research is ongoing which is directed at the cause, diagnosis, and treatment of this crippling disorder [2]. Animal experimentation has a long history in the field of diabetes research. To induce diabetes in animals, toxic chemicals such as streptozotocin [3] and alloxan [4] are popularly used. Transgenic rodent models of diabetes are gaining popularity. A major criticism of using rodents is that, due to their short life span, they may not develop the clinical complications seen in humans that have been diabetic for a number of years. Cats, dogs, swine, and nonhuman primates are occasionally used [5]. Rabbits are a potentially overlooked, manageable species for chronic diabetes experiments. They are considered lower on the phylogenetic scale than cats, dogs, swine, and nonhuman primates, and they have an average life-span of 5–8 years which is significantly longer than rodents.

Studies have demonstrated that rabbits possess characteristics desirable in a laboratory animal model including convenient size, longer life span, strain specific, have good temperaments, are easily handled and are relatively inexpensive [6]. We searched PubMed and in the past decade more than 1000 studies have been conducted using rabbits as a model of diabetes [7]. However, most of these experiments used rabbits that were diabetic for only several weeks to 2 months [8]. Studies of these durations have severely limited diabetic research capabilities as many complications of diabetes require years to develop [5]. In this study, we created a long-term rabbit model of type 1 diabetes mellitus with reasonably good health for experimental usage for one year or longer. This paper reports our management strategies and physical, biochemical, and histological findings of these rabbits. 

## 2. Methods

The study was conducted in accordance with the National Institutes of Health guidelines for the care and use of animals in research, and the protocol was approved by the Institutional Animal Care and Use Committee of the University of Louisville, an AAALAC accredited program. A total of 32 young (8–10 weeks, 2.13 ± 0.24 kg), male New Zealand white rabbits, Oryctolagus cuniculus (Myrtle's Rabbitry, Thompson Station, TN) were divided into two groups. The first group consisted of 20 animals with chemically induced type 1 diabetes mellitus; the second group consisted of 12 age-matched control (orthoglycemic) animals. Animals were singly housed in stainless steel caging with slotted floors (40 × 60 × 80 cm) in a temperature (16–22°C) and humidity (30–70%) controlled room on a 12 : 12 hour light : dark cycle. Cage pans were cleaned three times per week and cage racks were cleaned once weekly. Laboratory Rabbit Diet HF 5326 (LabDiet, PMI Nutrition International) supplemented with certified timothy hay cubes (Bio-Serv, Frenchtown, NJ) and filtered tap water was provided ad libitum through an automated watering system from arrival throughout the end of the study. Environmental enrichment consisted of a variety of fresh vegetables at least once weekly, dumbbells and stainless steel rattles (Bio-Serv, Frenchtown, NJ). Vendor microbiological monitoring health reports indicated that the rabbits were serologically negative for the following pathogens: CAR Bacillus, Encephalitozoon cuniculi, Pasteurella multocida, Treponema cuniculi, and Oral Papilloma Virus. The rabbits were also negative for Bordetella bronchiseptica, Salmonella, Klebsiella, Citrobacter, and Clostridium piliformis. Animals were free of internal and external parasites. Before any experimental manipulations were initiated the rabbits were allowed to acclimate for at least 7 days.

### 2.1. Induction and Management of Diabetes Mellitus

Rabbits were weighed weekly throughout the study and the weights were recorded. For the alloxan injection, rabbits were lightly anesthetized with ketamine hydrochloride 30 mg/kg and xylazine 3 mg/kg (IM). Artificial tears ointment (Butler Animal Health Supply, Dublin, OH) was applied to the surface of both eyes to prevent drying. Body temperature was maintained with a warm water circulating blanket (Gaymar T Pump, Gaymar Industries Inc., Orchard Park, NY). Heart rate, respiratory rate, body temperature, and SpO_2_ were monitored while the animals were under anesthesia and through recovery. Alloxan monohydrate (Sigma Aldrich Chemical, Saint Louis, MO, USA) was dissolved in sterile normal saline to achieve a concentration of 5% (W/V), and 100 mg/kg was immediately administered intravenously via the marginal ear vein over a period of 2 minutes with a 25 gauge butterfly catheter. The rabbits were allowed to recover from anesthesia. To avoid mortalities during the hypoglycemic phase, alloxan was administrated to nonfasted animals, and food and water were offered to animals immediately upon recovery after anesthesia. At 4, 8, and 12 hours following alloxan injection, 10 ml of glucose (5% W/V) was administered subcutaneously, and an oral solution of 20% glucose in tap water was provided via water bottle ad libitum for 1-2 days after confirmation of hypoglycemia (less than 70 mg/dl) to prevent hypoglycemic shock. During this period access to the automated watering system was restricted to encourage intake of the oral glucose solution. Rabbits whose blood glucose levels remained <300 mg/dl for more than one week following the initial injection of alloxan received a second dose of alloxan (100 mg/kg IV) to maintain a blood glucose level >300 mg/dl for the duration of the study. Blood glucose concentration was measured 1-2 times per day using a blood glucose meter (LifeSpan, Inc. Milpitas CA) for the first 4 weeks and once weekly thereafter in the morning. If morning blood glucose levels (BGL) were higher than 350 mg/dl, regular insulin (Novolin-R, Novo Nordisk Pharmaceuticals Inc. Princeton, NJ) was administered subcutaneously (SC) once daily with a 30 gauge needle. The insulin dose was adjusted according to the blood glucose level for the individual rabbit based on the following: (1) BGL < 400 mg/dl received 1 U/kg, (2) BGL = 400–500 mg/dl received 2 U/kg, (3) BGL = 500–600 mg/dl received 3 U/kg, and (4) BGL > 600 mg/dl received 4 U/kg.) In animals with BG levels > 500 mg/dl, a second insulin injection (half of the morning dose) was administered in the late afternoon. Blood and urine samples were collected for biochemical analysis weekly for two months then monthly until the end of experiment. Blood was collected from the central auricular artery with a 25 gauge needle. For urine collection, clean cage pans were placed under the cages without liners, and urine was collected for 24 hours.

### 2.2. Determination of Insulin Dose

Due to individual variation and sensitivity to insulin, the insulin dose for each rabbit was determined shortly after the induction of diabetes. A blood glucose curve was performed by administering a test dose of insulin (based on the aforementioned dosing rate) after measuring the blood glucose concentration in the morning. Blood glucose concentration was then measured every hour for 10 hours when it was clear that blood glucose levels were continuing to rise to obtain a glucose curve. If the lowest blood glucose was less than 50 mg/dl or higher than 200 mg/dl, a new glucose curve was obtained the next day by decreasing or increasing the insulin dosage until the best dose was obtained. The final insulin dose was determined when a glucose curve was obtained where the peak BGL was >350 mg/dl and the trough BGL was ≥50 mg/dl. If rabbits developed symptoms of illness (such as a loss of appetite, ptyalism, or severe weight loss) during the experiment, a new BG curve was obtained and a new insulin dose was implemented. 

### 2.3. Pinna Wound Healing Model

To evaluate the effect of long-term diabetes on wound healing, we created full thickness skin wounds on the ventral surface of the pinnae in both diabetic and nondiabetic age-matched rabbit groups at 2 weeks and one year using a minimally invasive surgical technique [9, 10]. Briefly, one hour before surgery, rabbits were injected subcutaneously with buprenorphine (0.03 mg/kg), and were anesthetized and monitored as described above. Under aseptic conditions, four circular full-thickness wounds were created on each ear with a 6-mm stainless steel punch. Prior to recovery a 25 **μ**g/hr Fentanyl transdermal patch (Duragesic, Ortho-McNeil, Raritan, NJ), was applied to the skin over the shoulders for analgesia. Sterile normal saline was used to dress all wounds. The wounds were then covered with Tegaderm (3M, St. Paul, MN, USA). Wound dressings were changed and digital photos were taken daily until the wounds were closed. Wounds were considered closed when they were totally reepithelialized [9, 10] and further confirmed by histology and morphometric analysis [11, 12] using Nikon Eclipse Ti microscope and Nikon Element software. 

### 2.4. Biochemistry Analysis

Plasma was obtained by centrifugation of blood samples at 2500 g for 20 min at 4°C, and was stored at −20°C until analysis. The activity of plasma lactate dehydrogenase (LDH), creatine kinase (CK), aspartate aminotransferase (AST), alanine aminotransferase (ALT), total protein, cholesterol, triglycerides (TG), blood urea nitrogen (BUN), and creatinine was assayed using a Roche Cobas Mira Plus chemistry analyzer (Roche Diagnostic System, Inc, Branchburg, NJ). Beta-hydroxybutyrate (BHBA) was also analyzed in some samples. All regents used in the analysis were purchased from CATACHEM (Bridgeport, CT, USA).

Urine samples were collected for 24 hours as previously described. The volume of urine collected was recorded, and its protein content was analyzed with a Roche Cobas Mira Plus chemistry analyzer. Ketones were analyzed by IDEXX VetLab (One IDEXX Drive, Westbrook, ME, USA). 

### 2.5. Preparation of Tissue Samples

At the end of 12 months, rabbits were euthanatized with 100 mg/kg pentobarbital (IV). Tissue samples were taken from the pancreas, liver, kidney, aorta, and pinna for histological studies. The samples were fixed in 4% buffered formaldehyde and embedded in paraffin. Six **μ**m slices were made from the paraffin blocks which were stained with hematoxylin and eosin and evaluated for histological changes.

### 2.6. Statistical Analysis

Results are reported as mean and standard deviation (SD). Student's *t*-test was used to compare the two groups and ANOVA was used for repeated longitudinal measurements with commercially available statistical software (GraphPad Software, Inc. San Diego, CA). A *P* value of <.05 was considered significant. 

## 3. Results

### 3.1. Animals

Of the 20 alloxan-treated rabbits, two died at 6 and 9 months secondary to renal failure. Four animals were used for a 2-week wound healing study, and 6 of the animals were used for a one-year wound healing study. The remaining 8 rabbits are still alive after 12 months.

### 3.2. Alloxan Dose and Hyperglycemia

Baseline nonfasting blood glucose levels were normal for all animals (124 ± 17 mg/dl). After the administration of alloxan there was a characteristic response in blood glucose level [4]. In the first 2 hours, blood glucose increased. The second phase, which was observed approximately 6 hours following alloxan injection, was a hypoglycemic phase which persisted for 24–48 hours. By that time the animals were alert, active, eating and drinking glucose water unassisted. It was determined that 24 hour continuous observation was not necessary. During our study, the blood glucose of some rabbits decreased to life threatening levels (less than 50 mg/dl). However, no animals died in the hypoglycemic phase of this study. The third phase was persistent hyperglycemia. Of the 20 alloxan-treated rabbits, blood glucose levels generally increased up to 300 mg/dl within 48 hours of receiving alloxan. After two weeks, the diabetic animals were divided into three groups according to their morning blood glucose levels: 300–400 mg/dl (*n* = 7), mild diabetic group; 401–500 mg/dl (*n* = 7), moderately severe diabetic group; greater than 501 mg/dl (*n* = 6), severe diabetic group. We found that animals in the mild group exhibited body weight increases rather quickly however their blood glucose levels decreased below 300 mg/dl 3–8 weeks after the first injection of alloxan, and a second injection of alloxan was required. Twelve animals required a second injection of alloxan to maintain persistent hyperglycemia (>300 mg/dl). An interesting finding is that the second injection of alloxan did not induce the characteristic hypoglycemic that was observed following the initial dose of alloxan. Therefore animals that received a second dose of alloxan did not require glucose supplementation. All animals that received a second dose of alloxan survived until the end of the experiment.

### 3.3. Insulin Dose and Glucose Concentration

In most of the rabbits, blood glucose concentrations followed a relatively predictable pattern. After insulin administration, blood glucose reached a trough (70–150 mg/dl) 2 hours after injection. By four hours after insulin BG levels began to rise and were >300 mg/dl within 6 hours and peaked the following morning before the next insulin injection. The mean time that rabbits were maintained in a hyperglycemic state was 16 hours. The mean time that rabbits were normoglycemic was 8 hours per day.

### 3.4. Body Weight

Baseline mean body weight of the rabbits was 2.13 ± 0.24 kg. Food intake was not measured in this study, however fecal output was assessed by husbandry staff daily and any decrease from normal production was brought to the attention of the study director and a clinical veterinarian. Although the blood glucose levels were very high, there was a tendency toward weight gain in all of the diabetic rabbits. There appeared to be a negative relationship between the highest blood glucose levels observed and body weight gain. At the end of 12 months, their body weights ranged from 3.12 to 4.18 kg, while the body weights of the age-matched nondiabetic rabbits ranged from 4.86–5.96 kg. The body weight increase of the diabetic rabbits was significantly lower than that of age-matched nondiabetic rabbits (*P* < .05).

### 3.5. Polyuria and Proteinuria

**Figure 1:**
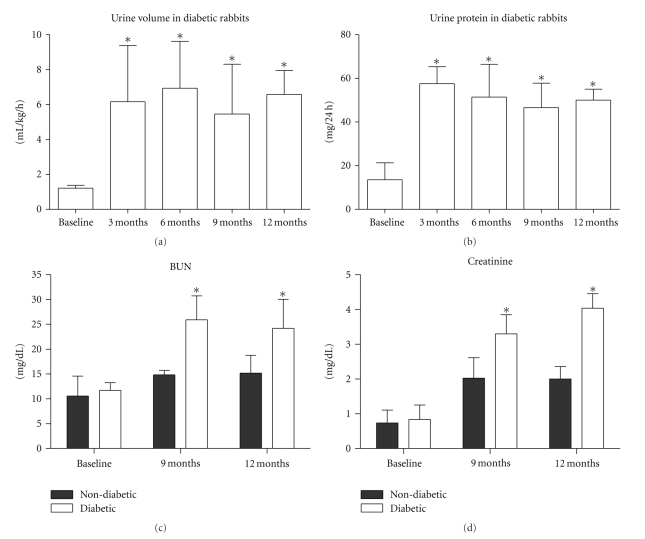
Changes in renal function. (a) and (b) there was a significant increase in urine and urine protein in diabetic rabbits as compared with baseline; (c) and (d) there was a statistically significant difference in blood urea nitrogen (BUN) and serum creatinine levels between the diabetic and age-matched nondiabetic rabbits at 9 and 12 months. (a) and (b) *P < .05  compared with baseline; (c) and (d) *P < .05 between diabetic and age-matched nondiabetic animals.

Both Polyuria and Proteinuria are characteristics of diabetes. Following diabetes induction in the rabbits, there were significant increases in urine volume (*P* = .0019, Figure 1(a)) and urine protein (*P* = .0004, Figure 1(b)) compared with the baseline.

### 3.6. Renal Dysfunction

Plasma levels of blood urea nitrogen (BUN) and creatinine increased following diabetes induction (Figures 1(c) and 1(d)). There were significant differences between the diabetic and age-matched nondiabetic rabbits at 9 months (*P* < .0001 for BUN and *P* = .0008 for creatinine) and 12 months (*P* = .0002 for BUN and *P* < .0001 for creatinine).

### 3.7. Hyperlipidemia

**Figure 2:**
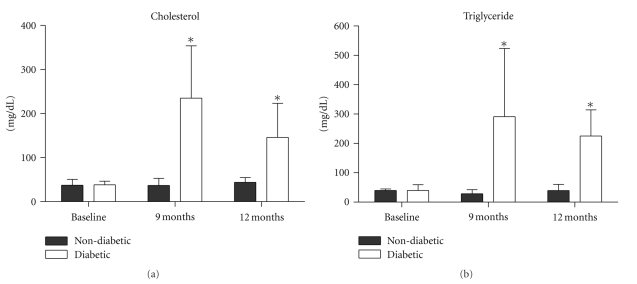
Hyperlipidemia: there were statistically significant differences in plasma cholesterol (a) and triglyceride (b) levels between the diabetic and age-matched nondiabetic rabbits at 9 and 12 months. *P < .0001 between the diabetic and age-matched nondiabetic rabbits.

Plasma cholesterol and triglyceride levels increased in all diabetic rabbits. There were significant differences between the diabetic and age-matched nondiabetic rabbits at 9 months (*P* < .0001 for cholesterol and *P* < .0001 for triglyceride) and 12 months (*P* < .0001 for cholesterol and *P* < .0001 for triglyceride, Figures 2(a) and 2(b)). 

### 3.8. Plasma Enzymes Levels

The majority of the plasma levels of ALT, AST, CK, and LDH were within normal range; however levels were elevated at times in individual rabbits. In addition, plasma total protein was within normal range although proteinuria was observed (Table 1).

### 3.9. Ketoacidosis and Urine Ketosis

**Figure 3:**
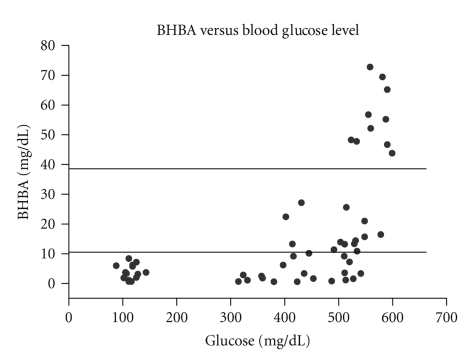
The relationship between blood beta-hydroxybutyrate (BHBA) and blood glucose concentrations. When the glucose levels increased above 500 mg/dl, the BHBA levels increased up to10 mg/dl (increase in ketone bodies) or greater than 40 mg/dl (ketoacidosis).

Diabetic ketoacidosis was demonstrated by increased blood beta-hydroxybutyrate (BHBA) [13]. When peak blood glucose levels were above 500 mg/dl, BHBA levels at times increased to more than 10 mg/dl or even greater than 40 mg/dl (ketoacidosis) (Figure 3). Ketones were also present in the urine of some severely diabetic rabbits whose peak blood glucose levels were greater than 500 mg/dl.

### 3.10. Wound Healing

**Figure 4:**
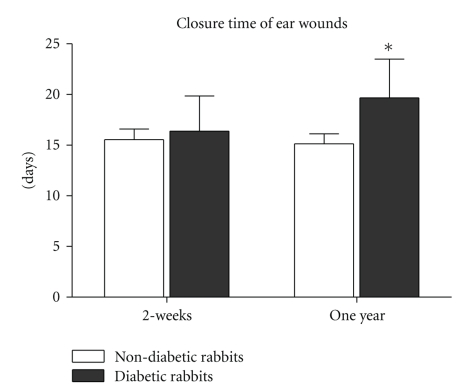
There were significant differences in closure times between one-year diabetic and age-matched nondiabetic wounds (*P* < .01). Healing times in 2-week diabetic rabbits were longer than the age-matched nondiabetic rabbits, however the difference was not statistically significant (*P* = .3865).

In rabbits wounded at two weeks following the induction of diabetes, wound closure time tended to be longer (16.5 ± 3.5 days) than the age-matched nondiabetic rabbits (15.6 ± 1.1 days), but the difference was not statistically significant (*P* = .3865). However, in rabbits that had been diabetic for one year, wound closure time (19.7 ± 3.7 days) was significantly longer than in age-matched nondiabetic rabbits (15.1 ± 1.0 days) (*P* < .01, Figure 4).

### 3.11. Other Complications

Throughout the study some animals exhibited anorexia, severe weight loss, ptyalism, weakness, and lethargy. These were caused by excessive hyper- or hypoglycemia, and all of them were treated by adjusting insulin dosages. Four rabbits suffered from slight anemia, with hemoglobin between 7–9 g/dl. The animals were not treated for anemia since it was not severe.

### 3.12. Pathologic Changes

**Figure 5:**
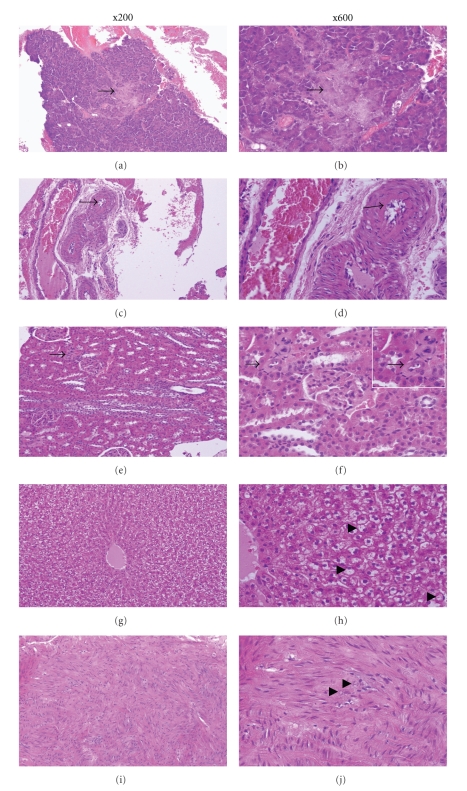
The pathological changes of a 12-month diabetic rabbit. (a) and (b) There is marked damage to beta-cells (arrow); (c) and (d) the microscopic cross-section of the artery in the pancreas shows a thickened vessel wall (arrow); (e) and (f) Hyaline arteriolosclerosis. The amorphous, homogeneous eosinophilic material is seen in the thickened vascular wall of afferent glomerular arteriole and the lumen is narrowed markedly (arrow). Glomerular atrophy is also present; (g) and (h) lipid accumulation in the cytoplasm of the hepatocytes appearing as vacuoles (hepatic fatty degeneration); (i) and (j) the microscopic section of the aortic media showing mild spot calcifications (arrow).

Histopathologic changes in animals that had diabetes for one year are shown in Figure 5. The pancreas showed marked beta-cell damage and thickened arterial walls. The kidney showed hyaline arteriolosclerosis. The amorphous, homogeneous eosinophilic material is seen in the thickened vascular wall of afferent glomerular arteriole and the lumen is narrowed markedly (arrow). Glomerular atrophy is also present. In the liver, there was lipid accumulation in the cytoplasm of the hepatocytes which appeared as vacuoles (hepatic fatty degeneration). The aortic media demonstrated mild spot calcifications in small areas.

## 4. Discussion

To our knowledge, this is by far the largest group of diabetic rabbits kept for one year or longer for scientific research. A long-term diabetic animal model has many potential benefits. Our experience has shown that, with careful management, alloxan-induced diabetic rabbits can be kept for one year or longer in reasonably good health. The biochemical and histological changes indicated well-developed diabetes. 

Many studies have been conducted in diabetic rabbits over short periods of time in the past several decades. However, short-term diabetic rabbits have severely limited the diabetic research capabilities because human diabetes lasts for decades and many diabetic complications take years to develop. A PubMed search revealed only a few reports in which diabetic rabbits were kept alive for 3–6 months for pharmacology or growth factor studies [14–18]. Only one article was found in which 3 rabbits were kept for 12 months to study the aortic intima-media of alloxan-diabetic rabbits [19]. Due to many medical complications, the management of long-term diabetic rabbits is labor intensive, expensive, and technically demanding which reduces the incentive to perform these studies over long periods of time. 

The toxin-induced diabetic rabbits were first administrated alloxan by Dunn and Mcletchie [20] who by studying the crush syndrome in rabbits investigated the effects of a series of uric acid derivatives including alloxan with regard to kidney damage. The animals which received alloxan became comatose after 12 hours, and were hypothermic with high blood urea and low blood glucose values. Shortly after the injection there was a marked hyperglycemia. Histological examination revealed early signs of expected kidney damage. An unexpected finding was a partial or total necrosis of the pancreatic islets. Other tissues showed no damage, and alloxan was gradually adopted to generate diabetes in animals. Over the course of our study, alloxan-induced diabetic animals exhibited classic symptoms of human diabetes, such as hyperglycemia, glucosuria, polydipsia, and polyuria, loss of body weight despite polyphagia, hyperlipemia, ketonuria, and acidosis. 

Following the administration of alloxan there is a characteristic response in blood glucose level [4]. In the first 2 hours, blood glucose rises. This transient hyperglycemia is thought to be due to sudden glycogen breakdown in the liver [21]. The reason for the breakdown of liver glycogen during this phase is unknown, but may be a secondary effect of epinephrine release. The second phase is a hypoglycemic phase which may be severe enough to lead to death if it is not prevented or treated with supplemental glucose [4, 22]. The hypoglycemia is due to a sudden outpouring of insulin from dying beta cells [21]. The hypoglycemia generally appears after 6 hours [23]. Hypoglycemia is more pronounced in fasted animals [24]. 

The susceptibility to both toxic and diabetogenic doses of alloxan varies widely not only in different species but also among animals of the same species [25, 26]. The range of the safe diabetogenic dose of alloxan in a particular animal is quite narrow and even light overdosing may be toxic and result in death. This loss is likely due to kidney tubular cell necrotic toxicity, in particular when high doses of alloxan are administered [27]. The dose of alloxan (100 mg/kg) utilized in this study is diabetogenic and less toxic. However, this dose does not necessarily produce stable diabetes in rabbits [28]. Sixty percent of the rabbits in this study required a second dose of alloxan to maintain persistent hyperglycemia, and all of these animals survived until the end of the experiment. The recovery from diabetes has been proposed to be the consequence of either a multiplication of beta cells that survive the initial alloxan injection or the formation of new beta-cells from the duct epithelium of the exocrine portion of pancreas [29, 30]. Another possible explanation is that there may have been a stress-induced hyperglycemia at the time of the initial alloxan injection initiated by handling of the animals. It has been shown that hyperglycemia can provide protection against alloxan-induced injury [31]. It has also been shown that male rabbits are more likely to experience stress-induced hyperglycemia than female rabbits [32] and this may have contributed to the high percentage of rabbits requiring a second dose of alloxan. In future studies we plan to measure the blood glucose level of the rabbits immediately prior to the administration of alloxan to be able to better predict those animals that may require a second dose of alloxan as well as increase the amount of handling that they receive during the acclimation period in an attempt to decrease the percentage of rabbits that require a second dose. Since untreated diabetics have higher complication rates than regulated diabetics, we chose to treat the rabbits in this study with insulin to increase the survival time of the rabbits, but we did not fully regulate the diabetes. Normal blood glucose levels were maintained a mean of 8 hours per day, and a hyperglycemic state was maintained for a mean of 16 hours per day. Therefore this model may more closely represent a model of poorly regulated diabetes because glucose fluctuation has been shown to cause more tissue damage than stable hyperglycemia [33]. 

Based on the severity of hyperglycemia, we divided the rabbits into three groups, those that had mild, moderately severe, and severe diabetes. Each group exhibited certain characteristic physiologic changes throughout the course of the study. In the mild diabetic group, body weights increased rather quickly, the biochemical values were usually in the normal range, the animals BG returned to levels <300 mg/dl and they required a second injection of alloxan. In the severe diabetic group, body weights increased very slowly and biochemical values were mostly abnormal including elevated BUN, anemia due to renal dysfunction [34], dyslipidemia [35], and at times ketoacidosis [36]. In the moderately severe diabetic group, the changes observed were a combination of the above two groups and their blood glucose levels were more manageable. 

In addition to hyperglycemia in the long-term diabetic rabbits, we observed other characteristics of diabetes. While all of the rabbits in this study gained body weight, the gain in the diabetic rabbits was less than the gain observed in the age-matched animals. Prior to alloxan administration, their body weights ranged from 2.0 to 2.5 kg. After one year, they only increased to 3.5–4.0 kg, a weight comparable to a normal 4-5-month old rabbit. However, the body weight of age-matched nondiabetic rabbits in this study increased to more than 5.0 kg after one year which is consistent with the normal growth rate provided by the vendor. The increase in body weight in this study, albeit small, appeared to be different from many of the previous studies in which diabetic rabbits lose weight. A large number of previous studies utilized rabbits that were 3-4 months of age and were considered to be young adults. Our finding of an increase in body weight is similar to that reported by Goseki et al. [16] who utilized young rabbits that were 10 weeks of age similar to those used in our study. Because body weight change is a reflection of whole body function, maintenance of appropriate body weight might be an important factor for the rabbits to survive long-term in the diabetic state. Plasma cholesterol and triglyceride levels increased in all diabetic rabbits in this study. These findings are in agreement with previously published studies [35]. We also found a few diabetic animals that suffered from anemia. These rabbits all had poor renal function, which is similar to clinical human patients due to a decrease in erythropoietin production by the damaged kidneys [37]. 

 The histological changes observed in animals kept for one year were typical of diabetic damage. In addition to pancreatic islet damage, microscopic cross-sections of the pancreatic arteries showed thickening of vessel walls which indicates atherosclerosis in this organ. In the aorta, microscopic sections of the aortic media demonstrated mild spot calcifications in small areas which are also indicative of atherosclerosis. Similar changes were also seen in the kidney, in which the afferent glomerular arteriole wall was markedly thickened and the lumen was narrowed. Glomerular atrophy was also present in the kidney. In the liver, lipid accumulated in the cytoplasm of the hepatocytes as vacuoles. All of these histological changes are consistent with those seen in long-term diabetics which may not be present in animals that are diabetic for shorter periods of time.

One of the most common complications of human diabetes is ulceration of the extremities, which has been subjected to numerous experimental studies. However, all of these studies suffer from several limitations that make it difficult to extrapolate animal results to clinical settings. One is the time during which the animal has been diabetic, and another is the etiology of diabetic ulcers. It is well known that human diabetic ulcers are the result of years, even decades of diabetes mellitus. Neuropathy, vasculopathy, immune dysfunction, and biochemical abnormalities all contribute to the development of chronic wounds in diabetic patients [38, 39]. To date, none of the animal models used for diabetic wound research have all of the same features of human diabetic wounds with the exception of hyperglycemia. The length of time that rabbits have been maintained in a hyperglycemic state in the majority of rabbit wound healing studies has been very limited, with the shortest being several days and the longest being two months. These short periods of persistent hyperglycemia simply cannot reproduce the changes observed in human diabetes. In order to test whether the time of hyperglycemia had any effect on wound healing, we compared wound healing times in diabetic rabbits and age-matched nondiabetic animals at two weeks and one year. To date little is known about the difference between wound healing in short-term hyperglycemia and long-term diabetes [8]. In the present study, short-term hyperglycemia (2 weeks) appeared to delay the healing process to a degree as compared to nondiabetic rabbits, but this difference was not statistically significant. However, in the one-year diabetic rabbits, the closure time of wounds was significantly longer than age-matched nondiabetic wounds. Our results appear to indicate that wound healing time is proportionate to the length of diabetic times. A state of systemic hyperglycemia, as seen in diabetes, may influence wound closure in numerous ways. Several hypotheses have been described in the literature such as the formation of glycation end products [40], hyperosmolarity [41], and altered insulin signaling in various ways [42]. However, hyperglycemia alone plays a limited role in the status of many nonhealing chronic wounds, while other pathophysiological changes take much longer to develop. If a rabbit's life span is 5–8 years, one year may be comparable to 15–20 years of human life, although such an approximation is an oversimplification. The biochemical and histological changes found in this study provide some evidence to mimic human diabetic changes; an animal model that is diabetic for a longer period of time than that which is more commonly published provides more features closer to human diabetic changes. 

In summary, with careful management which includes adaptive and repeated injections of alloxan, accurate adjustment of insulin dose, monitoring body weight and biochemical values, performing blood glucose curves, and adjusting insulin dosage as soon as animals exhibit abnormal symptoms, alloxan-induced diabetic rabbits can be kept for one year or longer in reasonably good health. Such a model may prove useful in scientific research on diabetes.

## Figures and Tables

**Table 1:** Changes of several serum biochemical measurements over 12 months in diabetic rabbits.

Index	Baseline	3 months	6 months	9 month	12 months
ALT (u/l)	16 ± 12	33 ± 16	28 ± 20	31 ± 17	26 ± 10
AST (u/l)	12 ± 6	24 ± 13	45 ± 30*	31 ± 18	25 ± 9
LDH (u/l)	48 ± 36	47 ± 26	65 ± 45	52 ± 26	78 ± 33
CK (u/l)	456 ± 140	494 ± 120	406 ± 193	465 ± 383	398 ± 279
TP (g/dl)	5.7 ± 1.5	5.4 ± 1.6	6.9 ± 1.9	5.6 ± 1.0	6.3 ± 1.3

ALT: alanine aminotransferase, AST: aspartate aminotransferase, LDH: lactate dehydrogenase, CK: creatine kinase, TP: total protein.

*P < .05 versus Baseline.

## References

[1] American Diabetic Association (2008). *National Diabetes Statistics Fact Sheet*.

[2] American-Diabetes-Association All about Diabetes. http://www.diabetes.org/about-diabetes.jsp.

[3] Tesch GH, Allen TJ (2007). Rodent models of streptozotocin-induced diabetic nephropathy (methods in renal research). *Nephrology*.

[4] Lenzen S (2008). The mechanisms of alloxan- and streptozotocin-induced diabetes. *Diabetologia*.

[5] Rees DA, Alcolado JC (2005). Animal models of diabetes mellitus. *Diabetic Medicine*.

[6] Mir SH, Darzi MM (2009). Histopathological abnormalities of prolonged alloxan-induced diabetes mellitus in rabbits. *International Journal of Experimental Pathology*.

[7] Sepici-Dincel A, Açikgöz Ş, Çevik C, Sengelen M, Yeşilada E (2007). Effects of in vivo antioxidant enzyme activities of myrtle oil in normoglycaemic and alloxan diabetic rabbits. *Journal of Ethnopharmacology*.

[8] Breen A, Mc Redmond G, Dockery P, O’Brien T, Pandit A (2008). Assessment of wound healing in the alloxan-induced diabetic rabbit ear model. *Journal of Investigative Surgery*.

[9] Wang J, Zhang Q, Wan R (2009). Intracellular adenosine triphosphate delivery enhanced skin wound healing in rabbits. *Annals of Plastic Surgery*.

[10] Wang J, Wan R, Mo Y, Li M, Zhang Q, Chien S (2010). Intracellular delivery of adenosine triphosphate enhanced healing process in full-thickness skin wounds in diabetic rabbits. *American Journal of Surgery*.

[11] Chvapil M, Gaines JA, Gilman T (1988). Lanolin and epidermal growth factor in healing of partial-thickness pig wounds. *Journal of Burn Care and Rehabilitation*.

[12] Gerharz M, Baranowsky A, Siebolts U (2007). Morphometric analysis of murine skin wound healing: standardization of experimental procedures and impact of an advanced multitissue array technique. *Wound Repair and Regeneration*.

[13] Guerci B, Tubiana-Rufi N, Bauduceau B (2005). Advantages to using capillary blood *β*-hydroxybutyrate determination for the detection and treatment of diabetic ketosis. *Diabetes and Metabolism*.

[14] Bozkurt NB, Pekiner C (2006). Impairment of endothelium- and nerve-mediated relaxation responses in the cavernosal smooth muscle of experimentally diabetic rabbits: role of weight loss and duration of diabetes. *Naunyn-Schmiedeberg’s Archives of Pharmacology*.

[15] Roy H, Bhardwaj S, Babu M (2006). VEGF-A, VEGF-D, VEGF receptor-1, VEGF receptor-2, NF-kappaB, and RAGE in atherosclerotic lesions of diabetic Watanabe heritable hyperlipidemic rabbits. *The FASEB Journal*.

[16] Goseki T, Ishikawa H, Nishimoto H (2006). Pharmacological vascular reactivity in isolated diabetic rabbit ciliary artery. *Experimental Eye Research*.

[17] Duzguner V, Kaya S (2007). Effect of zinc on the lipid peroxidation and the antioxidant defense systems of the alloxan-induced diabetic rabbits. *Free Radical Biology and Medicine*.

[18] Wadood N, Nisar M, Rashid A, Wadood A, Gul-Nawab, Khan A (2007). Effect of a compound recipe (medicinal plants) on serum insulin levels of alloxan induced diabetic rabbits. *Journal of Ayub Medical College, Abbottabad*.

[19] Richardson M, Hadcock SJ, Hatton BD, Winocour PD, Hatton MWC (1995). Proteoglycan alterations in the aortic intima-media of alloxan-diabetic rabbits: an ultrastructural and biochemical study. *Experimental and Molecular Pathology*.

[20] Dunn JS, Mcletchie NGB (1943). Experimental alloxan diabetes in the rats. *The Lancet*.

[21] Rerup CC (1970). Drugs producing diabetes through damage of the insulin secreting cells. *Pharmacological Reviews*.

[22] Duff GL, Brechin DJ, Finkelstein WE (1954). The effect of alloxan diabetes on experimental cholesterol atherosclerosis in the rabbit. IV. The effect of insulin therapy on the inhibition of atherosclerosis in the alloxan-diabetic rabbit. *Journal of Experimental Medicine*.

[23] Bell RH, Hye RJ (1983). Animal models of diabetes mellitus: physiology and pathology. *Journal of Surgical Research*.

[24] Spiegelman AR, Tuchman M (1955). Prevention of hypoglycemia during the induction of alloxan diabetes; the use of glucose and anti-hyaluronidase subcutaneously in the rabbit. *Diabetes*.

[25] Volk BW, Arquilla ER (1985). *The Diabetic Pancreas*.

[26] Zhao ZH, Watschinger B, Brown CD, Beyer MM, Friedman EA (1987). Variations of susceptibility to alloxan induced diabetes in the rabbit. *Hormone and Metabolic Research*.

[27] Pincus IJ, Hurwitz JJ, Scott ME (1954). Effect of rate of injection of alloxan on development of diabetes in rabbits. *Proceedings of the Society for Experimental Biology and Medicine*.

[28] Hadour G, Ferrera R, Sebbag L, Forrat R, Delaye J, De Lorgeril M (1998). Improved myocardial tolerance to ischaemia in the diabetic rabbit. *Journal of Molecular and Cellular Cardiology*.

[29] Cheţa D (1998). Animal models of type I (insulin-dependent) diabetes mellitus. *Journal of Pediatric Endocrinology and Metabolism*.

[30] Bencosme SA (1955). Cytology of islet cells in alloxan diabetic rabbits. *American Journal of Pathology*.

[31] Heikkila RE, Cabbat FS (1980). The prevention of alloxan-induced diabetes by amygdalin. *Life Sciences*.

[32] Monclús R, Rödel HG, Palme R, Von Holst D, De Miguel J (2006). Non-invasive measurement of the physiological stress response of wild rabbits to the odour of a predator. *Chemoecology*.

[33] Watada H, Azuma K, Kawamori R (2007). Glucose fluctuation on the progression of diabetic macroangiopathy—new findings from monocyte adhesion to endothelial cells. *Diabetes Research and Clinical Practice*.

[34] Al-Khoury S, Afzali B, Shah N (2007). Diabetes, kidney disease and anaemia: time to tackle a troublesome triad?. *International Journal of Clinical Practice*.

[35] Mathe D (1995). Dyslipidemia and diabetes: animal models. *Diabete et Metabolisme*.

[36] Harris GD, Fiordalisi I, Yu C (1996). Maintaining normal intracranial pressure in a rabbit model during treatment of severe diabetic ketoacidemia. *Life Sciences*.

[37] Singh DK, Winocour P, Farrington K (2009). Erythropoietic stress and anemia in diabetes mellitus. *Nature Reviews Endocrinology*.

[38] Gibran NS, Jang YC, Isik FF (2002). Diminished neuropeptide levels contribute to the impaired cutaneous healing response associated with diabetes mellitus. *Journal of Surgical Research*.

[39] Muangman P, Muffley LA, Anthony JP (2004). Nerve growth factor accelerates wound healing in diabetic mice. *Wound Repair and Regeneration*.

[40] Friedman EA (1999). Advanced glycosylated end products and hyperglycemia in the pathogenesis of diabetic complications. *Diabetes Care*.

[41] Yki-Järvinen H (1998). Toxicity of hyperglycaemia in type 2 diabetes. *Diabetes/Metabolism Reviews*.

[42] Porte D, Schwartz MW (1996). Diabetes complications: why is glucose potentially toxic?. *Science*.

